# An ominous triad: acute inferior ST-segment elevation myocardial infarction, right ventricular infarction, and ventricular septal rupture/dissection

**DOI:** 10.1093/ehjcr/ytad295

**Published:** 2023-07-08

**Authors:** Loukianos S Rallidis, Ioannis Xirogiannis, Konstantinos Papathanasiou

**Affiliations:** Second Department of Cardiology, University General Hospital, ‘Attikon’, 1 Rimini street, Chaidari, Athens 12462, Greece; Second Department of Cardiology, University General Hospital, ‘Attikon’, 1 Rimini street, Chaidari, Athens 12462, Greece; Second Department of Cardiology, University General Hospital, ‘Attikon’, 1 Rimini street, Chaidari, Athens 12462, Greece

A 63-year-old man was transferred from a rural hospital with cardiogenic shock caused by an inferior ST-segment elevation myocardial infarction (STEMI) (*Figure 1 Panel A*). He sought medical attention to the referral hospital due to sudden onset of severe dyspnoea, while his chest pain started 5 days ago. During the 2-h transportation, he was haemodynamically unstable, despite intravenous (i.v.) noradrenaline infusion, and on arrival, he was hypotensive (85/55 mmHg). Electrocardiogram showed inferior STEMI while right-sided leads (V3R–V6R) showed right ventricular infarction (RVI) (*Figure 1 Panel B*). Echocardiogram revealed inferior akinesis, severe right ventricular dilatation with poor function, ventricular septal rupture (VSR), and extensive longitudinal septal dissection with left to right communication through a ‘tunnel’ (*Figure 1 Panels C–F*, Supplementary material online, *Videos S1*, *S2*, and *S3* corresponding to *Figure 1 Panels C–F*). Due to progressing respiratory failure, he was put on mechanical ventilation. Afterwards, an intra-aortic balloon pump was inserted and coronary angiography revealed proximal total occlusion of right coronary artery (*Figure 1 Panels G* and *H*). The Cardiogenic Shock Team of our hospital was immediately activated and decided emergent surgical repair of the ventricular septum. Unfortunately, the patient suffered non-shockable cardiac arrest before reaching the operating room of our hospital and despite cardiopulmonary resuscitation, he died.

**Figure 1:**
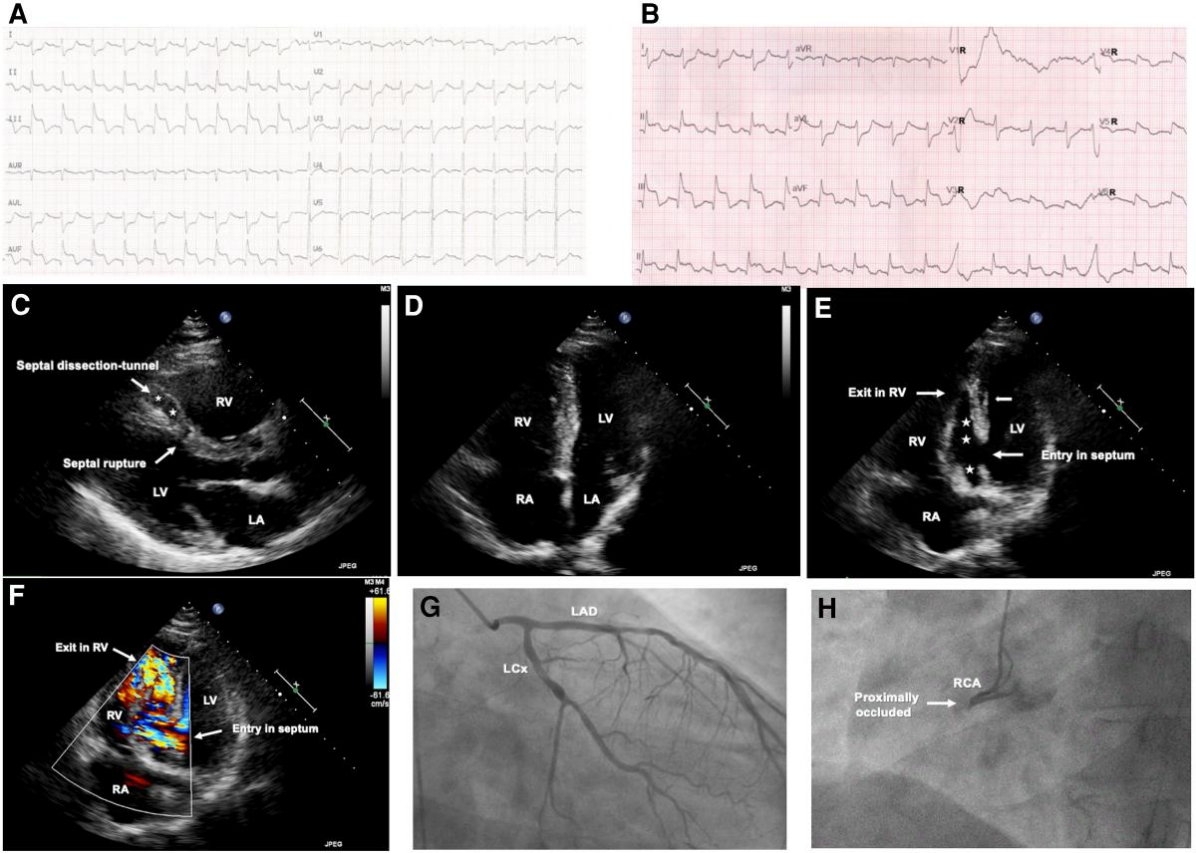
Electrocardiogram, echocardiogram and coronary angiography of the patient

Ventricular septal rupture complicating STEMI nowadays is rare while ventricular septal dissection (VSD) is extremely rare. In the setting of RVI, blood flow through VSR rapidly collapses RV. The decompensation is worse if the septum is dissected. This case shows the fundamental role of echocardiogram to illustrate early all the spectrum of complications of a non-reperfused timely inferior STEMI. The rare coexistence of inferior STEMI, RVI, and VSR/VSD forms a ‘deleterious’ triad.

## Supplementary Material

ytad295_Supplementary_Data

## Data Availability

The data underlying this article will be shared upon reasonable request to the corresponding author.

